# Longitudinal Impact of Frequent Geographic Relocation from Adolescence to Adulthood on Psychosocial Stress and Vital Exhaustion at Ages 32 and 42 Years: The Amsterdam Growth and Health Longitudinal Study

**DOI:** 10.2188/jea.JE20110141

**Published:** 2012-09-05

**Authors:** Kuan-Chia Lin, J. W. R. Twisk, Hui-Chuan Huang

**Affiliations:** 1 Department of Health Care Management, National Taipei University of Nursing and Health Sciences, Taipei, Taiwan; 2 Life-Course Epidemiology and Human Development Research Group, National Taipei University of Nursing and Health Sciences, Taipei, Taiwan; 3 Department of Epidemiology and Biostatistics, VU Medical Centre, Amsterdam and Department of Health Sciences, Faculty of Earth and Life Sciences, VU University, Amsterdam, Netherlands; 4 Department of Nursing, Cardinal Tien College of Healthcare & Management, Taipei, Taiwan

**Keywords:** geographic relocation, psychosocial stress, vital exhaustion, Amsterdam Growth and Health Longitudinal Study

## Abstract

**Background:**

We assessed mobility in different life stages over a 29-year period from adolescence through adulthood and its correlation with psychosocial stress and vital exhaustion at ages 32 and 42 years.

**Methods:**

Data were derived from the Amsterdam Growth and Health Longitudinal Study, an observational longitudinal study of 420 boys and girls from age 13 to 42 years. Measurements included cumulative frequency of geographic relocation (CFGR), psychosocial stress (measured by a Dutch scale of experienced stress, VOEG-13), vital exhaustion (measured by the Maastricht Questionnaire, MQ), demographics, socioeconomic status, and other background characteristics.

**Results:**

From 1976 to 2006, total CFGR was 3.56 ± 1.89 (range 0–13). Frequent geographic relocation during 2 life stages (age 22–32 years and 33–42 years) was significantly interrelated; however, this was not evident at age 13 to 21 years, which suggests a unique exposure to relocation during adolescence and youth. After adjusting for anticipated confounders, higher cumulative frequencies of residential changes during adolescence and youth were markedly associated with psychosocial stress and vital exhaustion at ages 32 and 42 years.

**Conclusions:**

Frequent geographic relocation during adolescence and youth was an indicator of psychosocial stress and vital exhaustion in the transition to middle adulthood. Further consideration of the pathways in this web of causation may aid in stress prevention and minimize negative consequences.

## INTRODUCTION

Geographic relocation can be a major life event in both adolescence and adulthood.^1^^–^^10^ It has been reported that during key developmental stages adolescents are particularly vulnerable to the stress of moving and that moving could intensify existing problems in children and the family nucleus.^1^^–^^8^ Acquiring new friendships may be challenging for adolescents still developing social skills. Psychologists theorize that people who move must deal with feelings of loss, fears of the unknown, and lessened parental attention, all of which may lead to emotional and behavioral problems.^11^^–^^13^

Moreover, after a life course transition, frequent geographic relocation from adolescence to adulthood may further affect an individual’s long-term context of residential stability.^14^ In turn, this might interact at the neighborhood, family, and individual levels in cumulative and compounding ways, with adverse effects on adult health. For instance, a high frequency of residential changes might be a useful marker of clinical behavioral and emotional risks. Because social context in different life stages may affect individual health trajectories, it has been hypothesized that the impact of frequent geographic moves begins in childhood and accumulates over the life course. Additionally, the impact of residential instability, sometimes combined with a felt lack of social support and coping mechanisms, may increase an individual’s psychosocial risk profile.

One construct closely related to established psychosocial risk profiles is psychosocial stress and vital exhaustion, which are states characterized by the presence of physical or psychological complaints, unusual fatigue, irritability, and demoralization. All these symptoms are typically attributed to prolonged psychological stress.^15^ Vital exhaustion, for instance, is characterized by fatigue, cognitive-affective depressive symptoms, sleep difficulties, and lack of concentration. In addition to current work and family situation, it is associated with several childhood stressors. In addition, significant associations were found with workload, household maintenance, childhood experiences with family conflicts, unemployment, financial problems, and adult experiences of marital status, unwanted childlessness, and issues of child education.^15^ In fact, vital exhaustion has been associated with certain behavioral risk factors and future increases in cardiovascular disease (CVD) risk and overlaps considerably with depression.^16^^,^^17^ However, its extent is less important than the fact that it contributes to prolonged psychological stress and is thus a public health burden in modern societies.

Although modern societies have become increasingly mobile, few prospective longitudinal studies have investigated the long-term association between geographic relocation and health from early adolescence to adulthood. Thus, little is known about how mobility in different life stages affects adult psychological/sociological mental health.

**Figure 1.**
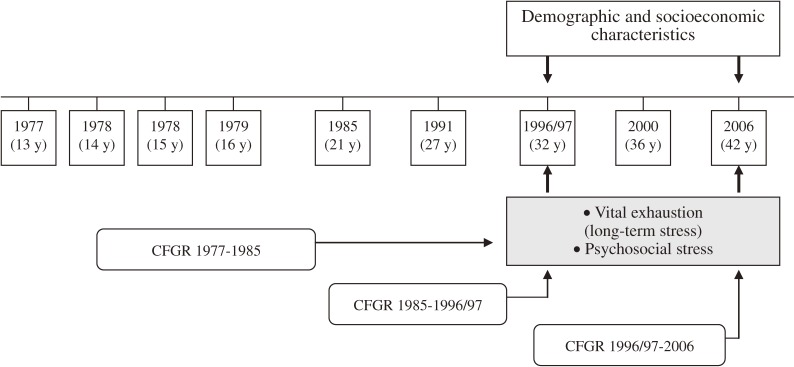
Illustration of longitudinal analyses in the study: longitudinal data on cumulative frequency of geographic relocation (CFGR) during different life stages and the impact of CFGR on vital exhaustion (long-term stress) and psychosocial stress in the transition to middle adulthood. The associations were adjusted for demographic and socioeconomic characteristics in adulthood.

The Amsterdam Growth and Health Longitudinal Study (AGAHLS) is a longitudinal study of repeated measurements performed on people aged 13 years or older over a period of 29 years.^18^^–^^24^ The measurements of AGAHLS were predominantly obtained during a medical check-up consisting of assessment of biological risk factors for CVD; physical fitness; psychological risk factors for cardiovascular disease; mental health; and lifestyle factors. At each measurement, details of geographic relocation were also assessed. Data for measures related to mental health in AGAHLS, the MQ, and VOEG were collected at age 32/33 years (period 1996/97) and age 42 years (period 2006), which thus provided an opportunity to observe how mobility during different life stages affects adult psychological/sociological mental health (Figure 1). The members of the present study provided longitudinal data on geographic relocation during different life stages and investigated the impact of such relocations on psychosocial stress (as measured by a Dutch scale of experienced stress, the VOEG-13) and vital exhaustion (long-term stress, as measured by the Maastricht Questionnaire; MQ) during the transition from adolescence to middle adulthood.

## METHODS

### Study design

The AGAHLS is a longitudinal study that began in 1977 with measurements of 420 boys and girls in 2 secondary schools (1 in Amsterdam and 1 in Purmerend, a suburb of Amsterdam). The study was approved by the medical ethics committee of Vrije Universiteit, Amsterdam, the Netherlands. Previous publications extensively describe the AGAHLS design.^18^^–^^24^ During the first 4 years of the study (1977, 1978, 1979, and 1980; age 13 through 16 years), 4 consecutive measurements were performed. Then, from 1985 to 2006, another 4 repeated measurements were completed: a sixth measurement in 1991 (mean age 27 years), a seventh in 1996/1997 (mean age 32 years), an eighth in 2000 (mean age 36 years), and a ninth in 2006 (mean age 42 years). To motivate participants to adhere to the longitudinal study, special measurements were taken during different age periods. For instance, during adolescence (1977–1985), interviews took place in classrooms and gymnasiums during regular school hours. After 1985, the participants had left school and were dispersed throughout the country. They were then asked to continue participation by paying annual visits to laboratories in Amsterdam. The present study analyzed data from 246 participants (112 males and 134 females; 122 from the Amsterdam group and 124 from the Purmerend group) who had provided information on measures of geographic relocation and remained in the study until age 42. Over the course of 23 years (from 1977 to 2000), about 35% of the participants dropped out. The overall drop-out rate during the 29-year period was 41.4% (246 participants from the present study after the 29-year period follow-up and 174 participants who did not continue the study until age 42). Selective dropout was investigated by testing differences in relevant characteristics between those who continued participation and those who had exited prematurely. Concerning the variables of interest, there were no statistical differences in baseline characteristics between the analyzed subsample of 246 participants and the other participants of the AGAHLS.^18^^–^^24^ However, for cumulative frequency of geographic relocation (CFGR), there may have been a dropout bias because the main reason for dropping out during adolescence (1977–1985) was leaving school (eg, parents moving away from Amsterdam and/or selection of the pupil by school authorities). Thus, there may have been an underestimation or nondifferential misclassification bias that undervalued risk.

### Geographic relocation

The primary independent variable of interest was CFGR,^6^^,^^8^ which was derived from the address and postal code of original residence upon study inception, the new address and postal code during every move from 1977 to 2006, the year in which the move took place, and the length of time since the last move (duration at current residence). To determine how mobility during different life stages affected adult psychosomatic/psychosocial development, we calculated CFGR (the main study independent variable) using 3 life stages: from 1977 to 1985 (participants at mean age 13–21 years), from 1986 to 1996/97 (mean age 22–32 years), and from 1998 to 2006 (mean age 32–42 years).^6^^,^^8^

### Outcome variables

For the AGAHLS, psychological/sociological characteristics were measured repeatedly during adulthood. For the present study, vital exhaustion and psychosocial stress were measured at age 32 and 42 years and are presented as the major outcome variables. Vital exhaustion was assessed with the Maastricht Questionnaire (MQ), a 23-item checklist of signs and symptoms of exhaustion.^15^^–^^17^^,^^25^^–^^27^ The MQ has good internal consistency, and Cronbach’s alpha for the Maastricht Interview on vital exhaustion was 0.89, indicating good reliability.^25^^–^^27^ This questionnaire was validated and proposed as a valid method to assess vital exhaustion in previous studies of the Dutch population.^26^^,^^27^ The Maastricht Interview on vital exhaustion was administered by a trained AGAHLS investigator, according to current standards. The interview consists of 23 questions on unusual fatigue, loss of energy, increased irritability, and feelings of demoralization, all scored as absent or present. The minimum score is 0 and the maximum score is 23.

In addition, to measure psychosocial stress, we used the 13-item version of the VOEG (a Dutch 21-item questionnaire on experienced health, which is designed to measure the tendency to somatize psychosocial stress and is used as a general stress indicator).^28^^–^^30^ The VOEG is a checklist for the presence of physical or psychological health complaints. The 13-item version (VOEG-13) was developed by Jansen and Sikkel^28^ and explains 95% of the original VOEG variance. The answer “yes” is coded as 1 and “no” as 0. The VOEG-13 consists of 13 dichotomous items that add up to a total list of health complaints ranging from 0 to 13. This abbreviated VOEG list was validated and shown to be a valid and reliable (Cronbach’s alpha = 0.83) indicator of psychosocial stress and general well-being.^29^^,^^30^ Because there is no conventional cutoff score, we used total score as an indicator of the amount of psychosocial stress experienced.

### Other parameters

Demographics (age, sex, marital status, employment details, number of adults in household, and number of children in household), socioeconomic status (level of educational attainment, personal and household income), level of urbanization, and other background characteristics upon last move in 1996/97 and 2006 were assessed to evaluate possible confounding effects.

### Statistical analysis

Data are summarized as the mean ± SD for continuous variables and as proportions for categorical variables. In view of skewness, the median and interquartile range (IQR) are also presented for CFGR. Differences in CFGR between groups at different life stages were tested with the Mann–Whitney U test and Friedman test, and, if applicable, Spearman correlation coefficients were estimated. To analyze the longitudinal relationship of CFGR with vital exhaustion and psychosocial stress, we used multiple linear regression (crude and adjusted) as a function of covariates (including demographics, socioeconomic status, level of urbanization status, and other background characteristics). Analysis and modeling strategies consisted of 2 steps. In crude models, CFGR in different life stages was treated as the major independent variable. Step 2 (adjusted models) then challenged the independent effect of CFGR by adding demographics, socioeconomic status, level of urbanization status, and other background characteristics as covariates. To estimate whether constant variance and linearity assumptions would hold, plots of the residual against its predicted value were created for regression diagnostics in all models. For all analyses, a 5% significance level was used. All statistical analyses were carried out using SPSS version 19 (SPSS, Inc., Chicago, IL, USA).

## RESULTS

**Figure 2.**
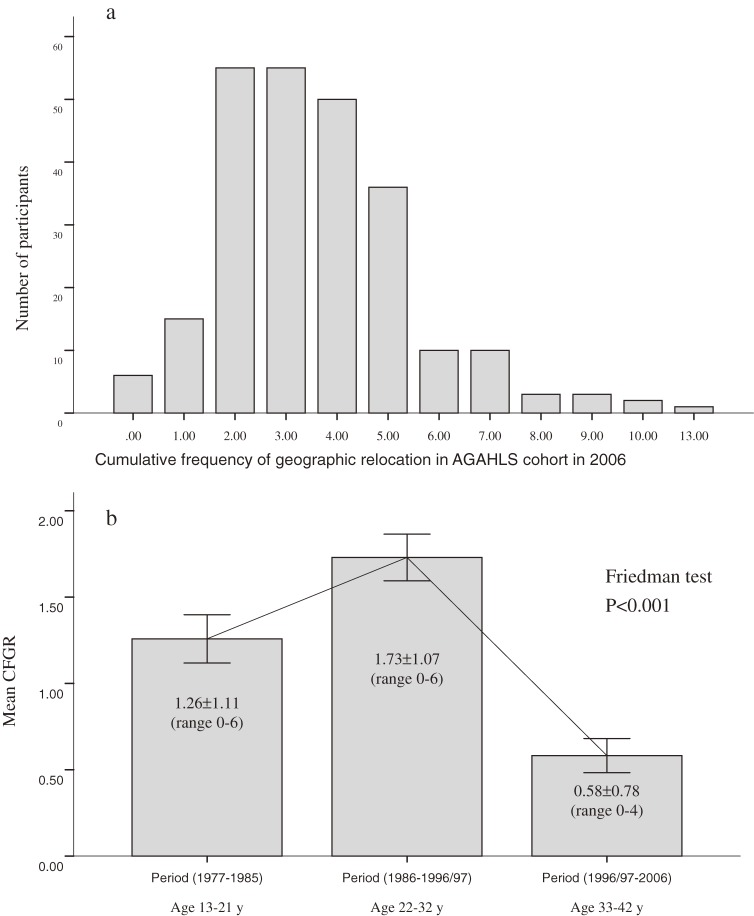
Distributions of (a) cumulative frequency of geographic relocation in the Amsterdam Growth and Health Longitudinal Study (AGAHLS) cohort in 2006 and (b) cumulative frequency of geographic relocation stratified by follow-up period in the AGAHLS cohort.

Table 1 shows the characteristics of the study population at ages 32 and 42 years. From 1977 to 2006, total CFGR was 3.56 ± 1.89 (median: 3; IQR: 3; range 0–13). Figure 2 shows the entire spectrum of CFGR and its progression from 1977 to 2006. There were significant differences in CFGR at different life stages, ie, from 1977 to 1985 (mean age 13–21 years), from 1986 to 1996/97 (mean age 22–32 years), and from 1998 to 2006 (mean age 32–42 years). When correlation analysis was used to study the interrelationships of CFGR during the 3 different life stages, a significant correlation was observed only between age 22 to 32 years and age 33 to 42 years (Spearman correlation coefficient = 0.28; *P* < 0.001).

**Table 1.** Characteristics of study population: demographics, socioeconomic status, and background data at mean ages 32 years (1997/97) and 42 years (2006)

Parameters corresponding to 1996/97 or 2006	Age 32/33 years (1996/97)		Age 42 years (2006)	
	
	*n* (%)	mean ± SD	*n* (%)	mean ± SD
Sex				
Male	112 (45.5)		112 (45.5)	
Female	134 (54.5)		134 (54.5)	
Cumulative frequency of geographic relocation		3.02 ± 1.56		3.59 ± 1.92
		(Medium: 3)		(Medium: 3)
		(IQR: 2)		(IQR: 3)
		(Range: 0–10)		(Range: 0–13)
Education level^a^				
Lower secondary education or below	14 (5.7)		14 (5.7)	
Higher secondary education	34 (13.8)		31 (12.6)	
Pre-university education	31 (12.6)		33 (13.4)	
University education or above	164 (66.7)		165 (67.0)	
Marital status				
Living alone	44 (17.9)		36 (14.6)	
Married	127 (51.6)		149 (60.6)	
Cohabiting	49 (19.9)		42 (17.1)	
Separated, Others	10 (4.1)		18 (7.3)	
Employment				
Unemployed	19 (7.7)		18 (7.3)	
Full-time	88 (35.8)		119 (48.4)	
Part-time	123 (50.0)		109 (44.3)	
Personal income (Euros)				
0–15 000	36 (14.6)		37 (15.0)	
15 000–30 000	59 (24.0)		55 (22.4)	
30 000–50 000	84 (34.1)		69 (28.0)	
50 000–100 000	28 (11.4)		64 (26.0)	
≥100 000	18 (7.3)		20 (8.1)	
Household income (Euros)				
0–30 000	NA		24 (9.8)	
30 000–50 000			62 (25.2)	
50 000–100 000			116 (47.2)	
≥100 000			42 (17.1)	
Number of adults in household		NA		1.82 ± 0.48
Number of children younger than 18 years in household		1.61 ± 0.74		1.67 ± 1.15

NA: Not available.

^a^Secondary education continues to approximately age 18. Pre-university education lasts 6 years in total and aims to prepare students for higher-level academic study.

Table 2 shows the results of longitudinal investigations of the relation of CFGR during different life stages with vital exhaustion and psychosocial stress. Crude analysis revealed that CFGR during the first life period (1977 to 1985; mean age 13–21 years) was strongly positively associated with psychosocial stress and vital exhaustion at age 32 years (β = 0.34, *P* = 0.020 for psychosocial stress; β = 0.38, *P* = 0.009 for vital exhaustion) and age 42 years (β = 0.44, *P* = 0.001 for psychosocial stress; β = 0.61, *P* = 0.003 for vital exhaustion). After adding demographic and socioeconomic characteristics as study covariates (adjusted analysis), CFGR history during age 13 to 21 years remained significantly positively associated with psychosocial stress and vital exhaustion at age 32 years (β = 0.38, *P* = 0.015 for psychosocial stress; β = 0.42, *P* = 0.004 for vital exhaustion) and age 42 years (β = 0.46, *P* < 0.001 for psychosocial stress; β = 0.72, *P* = 0.001 for vital exhaustion). In addition, the magnitude of the effect size (regression coefficient) was higher for the first life period and lower for both the second life period (from 1986 to 1996/97; mean age 22–32 years) and third life period (from 1998 to 2006; mean age 32–42 years) for both vital exhaustion and psychosocial stress.

**Table 2.** Parameter estimates and standard errors in statistical modeling to test the longitudinal relationship of cumulative frequency of geographic relocation (CFGR) with vital exhaustion and psychosocial stress conditions at ages 32 and 42 years in the AGAHLS

Frequency of geographic relocation	Vital exhaustion (long-term stress)						Psychosocial stress conditions					
	
	Age 32/33 years (1996/97)			Age 42 years (2006)			Age 32/33 years (1996/97)			Age 42 years (2006)		
			
	*B*	SE	*P*	*B*	SE	*P*	*B*	SE	*P*	*B*	SE	*P*
Crude model												
CFGR 1 (age 13–21 years)	0.38	0.09	0.009	0.61	0.21	0.003	0.34	0.14	0.020	0.44	0.13	0.001
CFGR 2 (age 22–32 years)	0.10	0.15	0.563	0.12	0.21	0.603	0.02	0.14	0.868	0.02	0.14	0.913
CFGR 3 (age 33–42 years)	—	—	—	0.14	0.30	0.648	—	—	—	0.02	0.19	0.918
Adjusted model^a^												
CFGR 1 (age 13–21 years)	0.42	0.08	0.004	0.72	0.20	0.001	0.38	0.12	0.015	0.46	0.10	<0.001
CFGR 2 (age 22–32 years)	0.13	0.19	0.503	0.15	0.21	0.487	0.02	0.15	0.798	0.02	0.13	0.810
CFGR 3 (age 33–42 years)	—	—	—	0.19	0.29	0.520	—	—	—	0.03	0.18	0.856

^a^Adjusted for demographic (sex, education level, marital status, and number of children and adults in household) and socioeconomic characteristics (employment status, personal and household income).

## DISCUSSION

On the basis of data from the AGAHLS, the current study provides epidemiologic cohort evidence that frequent geographic relocation from adolescence to youth affects adult psychological health. To the best of our knowledge, this study is one of the few longitudinal studies to address geographic relocation during different life stages on psychological/sociological health in adulthood.^6^^,^^7^^,^^31^^–^^34^ The AGAHLS started by following a group of healthy 13-year-old boys and girls until age 42 years. The longitudinal database therefore covers important periods during adolescence, young adulthood, and adulthood. The AGAHLS was conducted over an extended period of time and provides substantial epidemiological data on physiology and diseases relevant to human development from adolescence to adulthood.^18^^–^^24^ The duration of the AGAHLS study was nearly 30 years; however, excepting basic information and data on address changes, few variables related to social environment were measured.^18^^–^^20^ Thus, the present study was unable to adjust effectively for certain possible confounding variables during adolescence and youth, such as parental socioeconomic status, life events, learning achievements, and other factors contributing toward bias in causal inference. Nevertheless, because of these limitations, this study was able to adopt a more rigorous analytic framework. First, we attempted to correct for sociodemographic characteristics and the socioeconomic background of the AGAHLS participants throughout their growth into adulthood. Second, our analysis revealed that relocation experiences from adolescence to youth are independent of and statistically unrelated to relocation experiences upon entering adulthood. Using this foundation, multivariate analysis further revealed that frequent relocation was the only important factor in adulthood psychological health. Although numerous confounding variables during adolescence and youth have yet to be clarified, the present results emphasize the persistent effect of early-life relocation, a possible crucial indicator of underlying childhood and youth adversity that affects psychological development in adulthood.

The context for moving is diverse, eg, employment factors, perceptions of neighborhoods, housing tenure, and changes in family size and structure. These factors are linked in a complex manner to social and economic settings. From an epidemiologic point of view, the impact of residential instability during adolescence and youth on psychological/sociological health is of great importance in the transition to middle adulthood.^35^^,^^36^ The period comprising adolescence and youth is characterized by changes in social environment, specifically many closely spaced life changes, eg, residential mobility.^31^^,^^37^ Social networks develop and pervade multiple dimensions of residential stability, such as living in the same neighborhood and having a stable family environment. Evidence from previous studies suggests that the adolescent environment has a lasting impact on individual health, which is frequently conceptualized in terms of health inequalities beginning in adolescence and accumulating over the life course.^3^^–^^7^^,^^12^^,^^13^^,^^37^^,^^38^ Additionally, frequent geographic relocation significantly interacts at the neighborhood, family, and individual levels with cumulative and compounding consequences. Social perspectives at the family level consider the role of family stressors, including socioeconomic circumstances and the disruption of family structure.^32^^,^^39^ At an individual level, relocations involving school or neighborhood changes may be experienced as a life-event stressor that induces environmental adaptation. Residential stability may also increase adolescent connections to social and institutional networks, thereby offering the opportunity to develop strong social and community ties.^5^^,^^12^^,^^13^^,^^31^ Strong social networks in adolescence and youth may carry into adulthood, indirectly influencing an adult’s ability to establish supportive social networks. Another possible pathway for the effects of adolescent and youth environments on health at midlife is via their impact on health behavior over the life course.^1^^–^^5^^,^^33^^,^^37^ Therefore, a stable childhood environment may be related to superior health later in life.

As a consequence of the above findings, frequent geographic relocation has been cited as a stressful event and a cause of aberrant behavior among adolescents.^1^^–^^4^^,^^40^^,^^41^ Previous studies of youth showed that the correlation between these negative effects increased as more relocation was experienced.^12^^,^^13^ Adolescents who frequently relocate may be at increased risk for emotional problems because they must deal with the loss of both old friends and familiar environments and have to adapt to circumstances that they may not understand. It has also been suggested that frequent moving may itself be a marker of greater family problems and could lead to emotional stress in adolescents.^16^ Therefore, frequent geographic moves in adolescence and youth may be a marker of highly chaotic and stressed families, themselves characterized by emotional and unhealthy behaviors related to psychological distress.^1^^–^^5^^,^^32^^,^^33^ Parents who are preoccupied or exhausted with the physical and social demands of moving, eg, locating medical or child care in a new community and establishing new employment, may be less attuned to their child’s fears or needs. A previous study demonstrated that family moves and stress could be a potential source of stress in children and adolescents.^41^ Our findings support the hypothesis that sufficient stress in adolescence adversely influences health and persists into adulthood.^5^^,^^34^^,^^41^

In the current study, psychological/sociological conditions were represented by psychosocial stress (as measured by a Dutch scale of experienced stress; VOEG-13) and vital exhaustion (long-term stress, as measured by the Maastricht Questionnaire; MQ). Our analyses are notable because they identified how increased geographic relocation during adolescent and youth periods was associated with psychosocial stress and vital exhaustion in later life. The VOEG is a checklist for the presence of physical and psychological health complaints and measures the tendency to somatize psychosocial stress. In general, it is a reliable indicator of stress.^28^^–^^30^^,^^42^ Vital exhaustion is likely a broader concept that reflects not only continuous physiological overactivity, but also environmental overload. Thus, exhaustion is a marker of a person’s inability to cope with stress and his or her tendency toward excessive stress reactivity.^15^^–^^17^^,^^25^ According to the literature on cardiology and mental health, these 2 tendencies are related to loss of vigor, fatigue, and even depression.^43^ Exhaustion was also commonly reported among individuals who later developed myocardial infarction (MI) and cardiac death.^15^^–^^17^^,^^25^ Therefore, future studies must examine the extended impact of relocations during early life on later-life health conditions other than psychological conditions, eg, unhealthy behaviors, addiction, physiological diseases, and mortality.

In conclusion, on the basis of the study outcomes, we identified a longitudinal relationship of frequent geographic relocation during adolescence and youth with more symptoms of psychosocial stress and vital exhaustion in the transition to middle adulthood. Adolescent and youth experiences of residential instability or other mediators of health and development should therefore be considered with reference to medium- and longer-term psychological well-being. Further research that considers the pathways in this web of causation would be important to prevent or minimize negative health and psychological consequences.
